# Long-term effects of carbamazepine on bone-related biochemical markers in patients with epilepsy

**DOI:** 10.1186/s42494-026-00262-6

**Published:** 2026-06-02

**Authors:** Alqassem Y. Hakami, Azzam A. Althagafi, Abdulla S. Alkhayat, Belal A. Alturkistani, Hesham E. Mawar, Ibrahim H. Ibrahim, Haneen Abualhamail, Mohamed Eldigire Ahmed

**Affiliations:** 1 College of Medicine, King Saud bin Abdulaziz University for Health Sciences, Jeddah, 21423 Saudi Arabia P.O. Box 9515,; 2 King Abdullah International Medical Research Center, Jeddah, 22384 Saudi Arabia; 3 Pharmaceutical Services Department, King Abdulaziz University Hospital, Jeddah, 22252 Saudi Arabia; 4 College of Science and Health Professions, King Saud bin Abdulaziz University for Health Sciences, Jeddah, 21423 Saudi Arabia

**Keywords:** Epilepsy, Carbamazepine, Drug effect, Vitamin D, Calcium, Bone health

## Abstract

**Background:**

Carbamazepine (CBZ) is a commonly prescribed antiseizure medication frequently prescribed for epilepsy, trigeminal neuralgia, and mood disorders. However, CBZ is a strong inducer of cytochrome P450 enzymes, which can accelerate the catabolism of vitamin D and potentially alter bone metabolism. This study aimed to evaluate the effects of long-term CBZ administration on bone health in patients with epilepsy.

**Methods:**

An observational study involving 226 patients was conducted at King Abdulaziz Medical City in Jeddah, Saudi Arabia. We analyzed the pre- and post-treatment serum levels of vitamin D (nmol/L), alkaline phosphatase (U/L), sodium (mmol/L), and calcium (mmol/L) to assess biochemical changes associated with prolonged CBZ therapy.

**Results:**

The analysis of vitamin D revealed a significant decrease after CBZ treatment (mean ± SD= 36.6 ± 15.68 nmol/L) compared to pre-treatment (45 ± 23.25 nmol/L) with a 95% CI of -14.47 to -2.37 and *P =* 0.007. Additionally, following the administration of CBZ, serum levels of calcium and sodium were both significantly reduced with *P* < 0.001 and *P =* 0.019, respectively. However, alkaline phosphatase levels showed no significant change.

**Conclusions:**

The study outcomes suggest a potential association between long-term CBZ use and bone health, as supported by the presence of biochemical abnormalities. Furthermore, these findings underscore the importance of proactive clinical screening and bone-preserving interventions. Moreover, further studies are recommended to address the management of epilepsy patients with impaired bone health.

**Supplementary Information:**

The online version contains supplementary material available at 10.1186/s42494-026-00262-6.

## Background

Several studies have demonstrated that long-term carbamazepine (CBZ) use adversely affects bone health [1, 2]. Specifically, prolonged treatment with CBZ is associated with altered bone metabolism, reduced bone mineral density (BMD), and an increased risk of fractures. Epilepsy may further elevate fracture risk through mechanisms such as seizure-related falls, particularly in elderly patients [2]. Another study reveals that patients with epilepsy, particularly those receiving long-term antiseizure medications (ASMs), have a fracture risk ranging from twofold to sixfold higher than that of the general population [3]. Given the high prevalence of epilepsy and the chronic nature of ASM use, fracture-related morbidity represents a clinically significant burden for patients and healthcare systems.

Epilepsy is a chronic neurological condition characterized by the recurrent occurrence of unprovoked seizures [4]. The World Health Organization states that epilepsy impacts over 50 million individuals globally, with an estimated prevalence of 4–10 per 1,000 people, with more than 5 million new cases diagnosed annually [5, 6]. In Saudi Arabia, around 200,000 individuals are affected by epilepsy, placing a substantial burden on the country’s healthcare system [7].

ASMs are administered as the primary recommended choice of treatment to prevent seizure recurrence in epilepsy patients [8]. CBZ is a tricyclic ASM used primarily to manage focal seizures [4]. CBZ exerts its effect by inhibiting excitatory potential and decreasing synaptic transmission by altering voltage-gated sodium channels [9]. CBZ is metabolized in the liver and is also a potent cytochrome P450 enzyme inducer, which can lead to multiple drug–drug or drug–food interactions [10]. Typical side effects of CBZ include dizziness, nausea, vomiting, and drowsiness. CBZ may also lead to serious side effects, including CNS depression, nephrotoxicity, hepatotoxicity, and hyponatremia [9].

Although the exact mechanism is not well established, several studies have demonstrated theories regarding the osteopenic effects of CYP450-inducing ASMs. One such theory involves the dysregulation of vitamin D metabolism. CYP450-inducing ASMs, such as CBZ, may alter the activity of enzymes that metabolize vitamin D, thereby decreasing the levels of 1,25-dihydroxycholecalciferol [11, 12]. This effect may result in hyperparathyroidism as a secondary condition, leading to increased bone resorption and loss of bone mass [13].

A meta-analysis involving 20,787 healthy adults in Saudi Arabia revealed that vitamin D deficiency is prevalent in 63.5% [14]. Another report that included 13 studies (*N* = 24,399) showed the prevalence of vitamin D deficiency (< 50 nmol/L) among various age groups in Saudi Arabia to be approximately at 81% [13]. Additionally, the use of CBZ can potentially impact bone-related biomarkers, such as vitamin D, alkaline phosphatase, and calcium, in patients with epilepsy; however, most of the available evidence is derived from non-Saudi populations [15]. Therefore, the long-term effects of CBZ on vitamin D status and related bone biomarkers remain unclear in Saudi patients with epilepsy, particularly in the context of the high prevalence of vitamin D deficiency. To address this gap, the present study aimed to determine whether long-term CBZ use is associated with alterations in vitamin D status and bone biomarkers among patients with epilepsy in Jeddah, Saudi Arabia.

## Materials and methods

### Design and setting

This was a retrospective, single-center, observational pre–post study conducted at the Neurology Department of King Khalid Hospital, a tertiary care facility within King Abdulaziz Medical City–Jeddah (KAMC-J), Ministry of National Guard Health Affairs. The study period extended from May 2016 to December 2023. The study evaluated changes in biochemical markers before and after using CBZ administration. Due to the retrospective nature of this study, a convenience sampling method was employed, based on the availability of electronic medical records and laboratory data.

### Inclusion and exclusion criteria

**Fig. 1:**
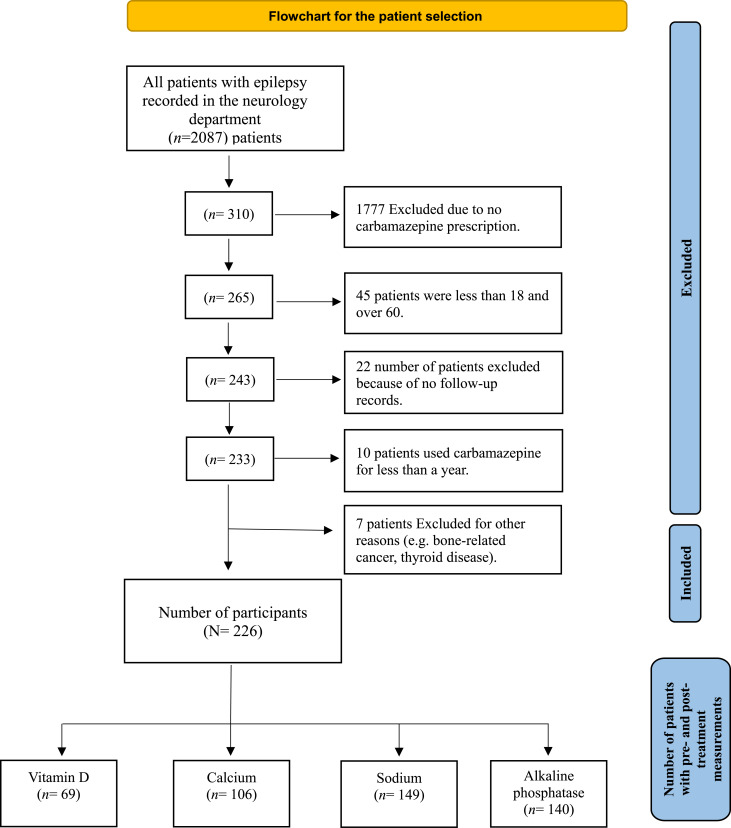
Flowchart demonstrating the selection process for study participants

Eligible participants were adult patients aged 18–60 years old with a diagnosis of epilepsy based on International League Against Epilepsy (ILAE) criteria [16], who received CBZ therapy for a minimum duration of one year. Patients were required to have at least one documented pre-treatment and one post-treatment measurement for at least one of the following biochemical markers: serum vitamin D, calcium, sodium, or alkaline phosphatase. Patients were excluded if CBZ was prescribed for any condition other than epilepsy, if follow-up data were unavailable, or if baseline biochemical markers before CBZ initiation were not available in the electronic medical record. Patients with documented conditions or using medications known to affect bone or mineral metabolism before or during CBZ therapy were excluded (e.g., advanced chronic kidney disease, chronic liver failure, primary hyperparathyroidism, active malignancy involving bone, or long-term systemic corticosteroid therapy). A detailed list of excluded conditions and medications is provided in Supplementary Table S1. The use of other antiseizure medications was not an exclusion criterion but was recorded and accounted for as a potential confounder in the statistical analysis. After screening 2,087 medical records, 226 patients met the eligibility criteria. Due to variability in laboratory testing as a part of routine medical care, the available sample size differed by biochemical parameter; therefore, analyses were conducted using separate subgroups for each biochemical marker: vitamin D, calcium, sodium, and alkaline phosphatase. Figure 1 illustrates the participant selection process.

### Data collection

Data were extracted from the hospital electronic medical record system (BestCare 2.0) by five trained data collectors using a standardized electronic data collection form. To ensure consistency, all data collectors were trained on the study protocol, and standardized variable definitions were used throughout the data extraction process. Baseline variables included age, sex, body mass index (BMI), duration of CBZ therapy, CBZ dose, comorbidities, and concomitant use of other antiseizure medications. Laboratory parameters collected included serum 25-hydroxyvitamin D (nmol/L), total serum calcium (mmol/L), serum sodium (mmol/L), and total alkaline phosphatase (U/L). The pre-treatment measurements were defined as values obtained within three months before CBZ therapy initiation. Post-treatment measurements were defined as the most recent laboratory values and had to be obtained at least 12 months after CBZ initiation. Due to the retrospective nature of the study, the timing of laboratory assessments varied across patients and was dependent on routine clinical practice.

### Data analysis

Statistical analyses were performed using JMP Pro version 14 (SAS Institute Inc., Cary, NC, USA). Categorical variables were presented as frequencies and percentages, while continuous variables were presented as means and standard deviations. The Shapiro–Wilk test was used to assess the normality of continuous variables. For pre–post comparisons, the paired-sample *t*-tests were used for normally distributed variables, and the Wilcoxon signed-rank test was utilized when normality assumptions were not met. Multiple linear regression analyses were conducted to investigate factors associated with the change in serum vitamin D and calcium levels between pre- and post-treatment measurements. Independent potential variables were selected based on clinical relevance, including age, sex, BMI, duration of CBZ therapy, CBZ dose, comorbidities, and concomitant use of other antiseizure medications. Due to the limited sample size for some biochemical parameters, regression analyses were considered exploratory. Statistical significance was defined as a two-sided *P*-value ≤ 0.05.

## Results

**Fig. 2:**
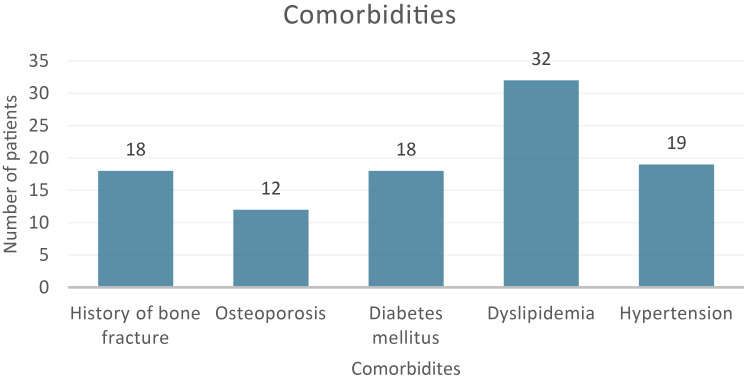
Distribution of comorbidities among the study participants

Among the 226 patients, 116 were female (51.33%), and 110 were male (48.67%). The average age was 38 years, and the mean duration of CBZ therapy was approximately 50.8 months with a total daily dose averaging 648 mg (mean ± SD = 648 ± 299.43), as shown in Table 1. Furthermore, out of 226 patients included in the study, dyslipidemia was the most frequently observed comorbidity in 32 patients (14.16%) followed by hypertension in 19 patients (8.41%). The distribution of comorbidities among the study participants is illustrated in Fig. 2. Statistical analysis of vitamin D subgroup showed a significant decrease after the administration of CBZ therapy (mean ± SD = 36.6 ± 15.68 nmol/L) compared to before treatment (mean ± SD = 45 ± 23.25 nmol/L), and a *P*-value of 0.007. In the calcium subgroup, the average serum calcium level was significantly lower after the administration of CBZ (2.2 mmol/L) compared to pre-treatment mean (2.3 mmol/L) (*P*-value of < 0.001). Furthermore, statistical analysis of the sodium serum level showed a statistically significant decrease following CBZ therapy, as indicated by a *P*-value of 0.019. Statistical analysis indicated that there was no significant change in serum levels of alkaline phosphatase following CBZ therapy (more details in Table 2).

**Table 1:** Participant demographics and clinical characteristics

Demographic characteristics Parameters	Patient with epilepsy (*N* = 226) Mean ± SD (%)
Age (years)	38 ± 11.13
Female sex (frequency%)	116 (51.33%)
Male sex (frequency%)	110 (48.67%)
BMI (kg/m^2^)	29.4 ± 8.26
Length of taking medication (months)	50.8 ± 29.78
Average total daily dose (mg)	648 ± 299.43
**The use of another antiseizure medications**	***n*** **(%)**
As monotherapy	83 (36.72%)
As dual therapy	81 (35.84%)
As triple therapy	37 (16.37%)
Concomitant antiseizure medications*	
Valproic acid	27 (11.95%)
Oxcarbazepine	12 (5.31%)
Ethosuximide	1 (0.44%)
Topiramate	31 (13.72%)
Levetiracetam	95 (42.04%)
Lamotrigine	29 (12.83%)
Lacosamide	29 (12.83%)
Phenytoin	11 (4.87%)

*Patients may have received more than one concomitant antiseizure medication. SD= Standard deviation, BMI= Body mass index, *n*= number of participants

**Table 2:** Biochemical parameters before and after CBZ administration

Variable	*N*	Before CBZ (Mean ± SD)	After CBZ (Mean ± SD)	95% CI	*t* statistic	*P*-value
Vitamin D (nmol/L)	69	45 ± 23.25	36.6 ± 15.68	-14.47 to -2.37	-2.775	0.007*
Calcium (mmol/L)	106	2.3 ± 0.10	2.2 ± 0.13	-0.11 to -0.05	-5.44	< 0.001*
Sodium (mmol/L)	149	137.4 ± 2.6	136.7 ± 3.33	-1.41 to -0.77	-2.37	0.019*
Alkaline phosphatase (U/L)	140	106.5 ± 68.18	111.4 ± 68.63	-4.43 to 14.29	1.04	0.299

CBZ= carbamazepine, SD= stander deviation, CI= Confidence interval, * Statistically significant at *P* ≤ 0.05

Multiple linear regression analysis examined the association between factors and the change in the predictor’s vitamin D and calcium levels from baseline to post-CBZ therapy, as detailed in Tables 3 and 4. In Table 3, the overall model was statistically significant, indicating that the independent variables included accounted for 55.6% of the variability in the difference in vitamin D levels. Among the predictors, age positively contributed to the increase in vitamin D, with unstandardized coefficients of 0.867 (*P* = 0.01). Additionally, dyslipidemia was associated with significantly lower vitamin D levels by 23.45 nmol/L (*P* = 0.018). Regarding ASMs, topiramate (*P* = 0.001) and phenytoin (*P* = 0.001) were significantly associated with reduced vitamin D levels. Conversely, lamotrigine positively correlated with vitamin D levels (*P* = 0.019). However, other variables did not show statistically significant associations with vitamin D levels (*P* > 0.05). In Table 4, no significant predictors were identified for differences in calcium levels.

**Table 3:** Multiple linear regression analysis of change (*Δ*) in serum vitamin D levels

	β	*t* statistic	*P*-value	95% CI for β
Age	0.867	2.699	0.010*	0.222 to 1.513
Duration	0.272	1.842	0.072	-0.025 to 0.570
Total dose	0.004	0.463	0.646	-0.015 to 0.024
Osteoporosis	-14.503	-1.390	0.171	-35.464 to 6.457
Dyslipidemia	-23.458	-2.444	0.018*	-42.749 to -4.167
Topiramate	-28.189	-3.446	0.001*	-44.629 to -11.750
Lamotrigine	26.141	2.416	0.019*	4.399 to 47.884
Phenytoin	-58.442	-3.563	0.001*	-91.403 to -25.482

Dependent variable: change (*Δ*) in vitamin D level. *R²* = 0.556; *F* = 3.406. *Statistically significant at *P* ≤ 0.05, CI= Confidence Interval

**Table 4:** Multiple linear regression analysis of change (*Δ*) in serum calcium levels

	β	*t* statistic	*P*-value	95% CI for β
Age	0.002	1.585	0.117	-0.001 to 0.005
Duration	0.000	− 0.324	0.747	-0.001 to 0.001
Total Dose	0.000	-1.806	0.074	0.000 to 0.000
Osteoporosis	-0.109	-1.654	0.102	-0.240 to 0.022
Dyslipidemia	− 0.052	-1.039	0.302	-0.152 to 0.048
Topiramate	− 0.001	− 0.019	0.985	-0.98 to 0.097
Lamotrigine	0.032	0.658	0.512	-0.65 to 0.130
Phenytoin	0.005	0.067	0.947	-0.144 to 0.154

Dependent variable: change (*Δ*) in calcium level. *R²* = 0.348; *F* = 2.583. *Statistically significant at *P* ≤ 0.05

## Discussion

Despite the emergence of second and third-generation ASMs, CBZ remains one of the primary options or an alternative in certain cases for treating focal and primary generalized tonic-clonic seizures [10]. The effects of CBZ on bone health and the endocrine system are not well understood [17–19]. Furthermore, numerous epilepsy patients need prolonged ASM treatment, placing them at greater risk for negative metabolic side effects from the medications [20].

The present study investigated the impact of long-term CBZ treatment on vitamin D, calcium, sodium, and alkaline phosphatase levels in a Saudi population. The findings suggest a possible deleterious effect of CBZ on these markers, showing reductions in vitamin D, calcium, and sodium levels; however, alkaline phosphatase levels remained largely unchanged. To our knowledge, this is the first study in Saudi Arabia to evaluate long-term CBZ effects on these biochemical markers. Our study used a within-patient design, which reduces interindividual variability and control group bias. Additionally, the relatively large sample size and assessment of polytherapy effects strengthen the clinical relevance of our findings [21–25].

Various theories have been proposed to explain how ASMs can lead to bone diseases. One of the principal mechanisms involves the modification of vitamin D metabolism. ASMs that induce cytochrome P450 enzymes decrease levels of active vitamin D, which, in turn, reduces calcium absorption from the gut and increases alkaline phosphatase levels. The decrease in calcium levels elevates circulating parathyroid hormone, subsequently increasing calcium mobilization and bone mass loss [11, 12, 26]. However, this mechanism does not account for all the effects described on bone health associated with ASMs. For example, some studies have found that valproic acid, a CYP450-inhibitor ASM, can decrease BMD levels [27, 28]. Some studies have linked the reduction in vitamin D and BMD in patients receiving ASMs to genetic variations, particularly in the vitamin D receptor genes [29, 30]. Because there is insufficient evidence regarding the effect of CBZ on bone metabolism in our region, addressing this knowledge gap is particularly important.

Both vitamin D and calcium levels significantly decreased with CBZ treatment, and these results align with several studies [2, 15, 21]. However, other studies have shown contrasting outcomes [25, 31]. Factors such as age, genetic variations, diet, exercise, and sunlight exposure may have contributed to the variability observed in these studies. A noteworthy finding in the present research is that, despite not excluding patients on vitamin D supplements, the overall levels of vitamin D decreased in patients undergoing CBZ treatment. This could be due to a lack of awareness regarding the effects of ASMs on bone health or patient noncompliance with taking supplements.

Notably, in this study, the use of topiramate or phenytoin in combination with CBZ was associated with greater reductions in vitamin D levels, while the use of lamotrigine with CBZ was a significant predictor of increased vitamin D levels. Consistent with our findings, previous research reported that phenytoin monotherapy can significantly decrease vitamin D levels [32, 33], whereas lamotrigine does not [21]. Interestingly, a prior study reported that topiramate monotherapy does not alter vitamin D levels [34]. However, recent evidence suggests that when combined with valproic acid, topiramate may significantly reduce vitamin D levels, similar to results with CBZ [35]. Studies investigating the effect of ASM polytherapy on bone health are scarce, and future research that confirms these findings is recommended due to the small number of patients taking these medications in our study.

The reduction in vitamin D levels in this study was more pronounced in patients with dyslipidemia. This finding aligns with several cross-sectional studies which have demonstrated a strong association between vitamin D deficiency and dyslipidemia [36, 37]. Dyslipidemia frequently coexists with insulin resistance and chronic low-grade inflammation, and these conditions may promote sequestration of vitamin D in adipose tissue and increase hepatic turnover of vitamin D metabolites [38]. In addition, dyslipidemia itself is also associated with elevated levels of parathyroid hormone [39]. These factors may partly explain why patients with dyslipidemia experienced a greater reduction in vitamin D with CBZ therapy.

Although several studies reported no significant change in alkaline phosphatase levels with CBZ treatment [25, 40], a meta-analysis conducted by Zhang et al. [15] revealed a significant increase in alkaline phosphatase levels due to CBZ. The extent of this increase, however, was particularly greater in the pediatric group. In contrast to the meta-analysis results, alkaline phosphatase levels did not significantly increase in our study. The variability of results among studies may stem from data derived from patients of different age groups and with varying levels of inactivity, sun exposure, and recurrent infections [40].

Hyponatremia has been reported as a potential risk factor for fractures and falls [41, 42]. Two mechanisms may explain this association: first, hyponatremia might induce mild cognitive impairment, which can lead to unsteady gait and falls [43]. Another suggested mechanism is that hyponatremia can increase bone resorption directly. Low extracellular sodium levels can stimulate osteoclastogenesis and bone resorption. Additionally, patients with hyponatremia may have elevated levels of arginine vasopressin, which can stimulate bone-resorbing osteoclasts and inhibit bone-forming osteoblasts [44]. In accordance with prior research [45], sodium levels significantly decreased following CBZ treatment in our study. One possible mechanism for the reduction in sodium levels associated with CBZ treatment is related to the drug’s antidiuretic effect. CBZ can directly affect the collecting ducts of the kidneys, which ultimately increases osmotic water absorption and decreases serum sodium levels [46].

While CBZ treatment was associated with statistically significant reductions in calcium, vitamin D, and sodium, the absolute changes should be interpreted in the context of clinical reference ranges as most post-treatment values remained within the reference range. Nevertheless, the reduction in these parameters might result in clinically significant outcomes for patients who were reported to be deficient during baseline measurement, potentially increasing the risk of bone fracture. For example, a decrease in calcium could result in hypocalcemia, leading to neuromuscular symptoms and increased susceptibility to fractures [47, 48], while further reductions in vitamin D levels may exacerbate impaired bone mineralization and fracture risk [49]. Future analyses stratifying patients by baseline vitamin D levels may help clarify its relationship with the clinical outcomes.

Although this study possesses notable strengths, it is essential to acknowledge its limitations. First, the study assessed biomarkers only without measurements of BMD, incidence of fracture, or other markers of bone turnover, such as osteocalcin and parathyroid hormone, as these are not part of the routine investigations conducted in the clinic for these patients. Therefore, the findings reflect associations with bone-related markers rather than definitive changes in bone health. Although the average doses of CBZ for all participants were calculated, some patients’ doses were adjusted based on their epilepsy control status. These variations could have influenced the relationship between total daily CBZ dose and the extent of changes in calcium and vitamin D levels. Moreover, the study inclusion criteria did not exclude patients using vitamin D supplements as this is an observational study and patients might be on the supplements without the proper drug name or dose documentation in the hospital electronic medical record. Thus, the observed impact of CBZ on vitamin D levels in our study may have been lower than the actual effect. Furthermore, convenience sampling and patient subgrouping based on the availability of laboratory data may have introduced selection bias, which could potentially limit the generalizability of the results. Additionally, pregnancy status, menopausal status, and sun exposure could not be reliably assessed due to the study’s retrospective observational design, which can inherently limit the ability to establish causality. Finally, residual confounding from concomitant ASMs and comorbidities may have influenced the observed changes in bone-related biochemical markers, despite our pre- and post-CBZ treatment analyses. To that end, further prospective research measuring bone-related biochemical markers, including parathyroid hormone and osteocalcin, BMD, and fracture rates, in patients receiving ASM treatment is warranted.

## Conclusions

The results of this study suggest a potential association between long-term CBZ use in adults and biochemical abnormalities, as evidenced by decreases in serum vitamin D, calcium, and sodium levels. These findings are likely to be clinically relevant, as vitamin D deficiency, hypocalcemia, and hyponatremia are linked to changes in bone metabolism leading to decreased bone strength, increased fall risk, and a higher incidence of fractures in patients undergoing prolonged CBZ therapy for epilepsy. This study provides novel regional evidence by being among the first in the region to evaluate these findings on long-term CBZ use using a pre- and post-treatment design. Routine measurements of serum vitamin D, calcium, and sodium levels are generally recommended as part of the routine care of patients with epilepsy to facilitate early intervention and supplement prescription when indicated. In addition, patients should be encouraged to adopt a bone-preserving lifestyle, including regular physical activity and a healthy balanced diet.

## Electronic Supplementary Material

Below is the link to the electronic supplementary material.

Supplementary Material 1

## Data Availability

The data used in this study is available from the corresponding author on reasonable request.

## Abbreviations

ASMs: Antiseizure medications
BMD: Bone mineral density
BMI: Body mass index
CBZ: Carbamazepine
