# Supplementary material for: Long-term effects of carbamazepine on bone-related biochemical markers in patients with epilepsy
Source: Acta Epileptol. 2026 Jun 2;8:22. doi: 10.1186/s42494-026-00262-6 (PMC13227868; doi:10.1186/s42494-026-00262-6)
Supplement: Supplementary file 1 — Supplementary Material 1 [file 42494_2026_262_MOESM1_ESM.docx]

| Supplementary Table S1. Details on conditions and medications affecting bone or mineral metabolism as exclusion criteria |
| --- |
| \| Category \| Condition / Medication \| \| --- \| --- \| \| Endocrine \| hyperparathyroidism \| \|  \| Hyperthyroidism \| \| Renal \| Chronic kidney disease stage 4–5 (eGFR <30) \| \| Hepatic \| Chronic liver failure \| \| Oncology \| Active malignancy with bone metastasis \| \| Medications \| Bisphosphonates / Denosumab / Teriparatide / Long-term systemic corticosteroids \| \| Gastrointestinal \| Malabsorption syndromes (celiac, IBD with malabsorption) \| \| Rheumatologic \| Osteogenesis imperfecta, Paget disease \| |
